# Variable selection in mixture cure models using elastic net penalty: application to COVID-19 data

**DOI:** 10.1371/journal.pone.0320521

**Published:** 2025-05-07

**Authors:** Aluwani Ramalata, Akim Adekpedjou, Maseka Lesaoana

**Affiliations:** 1 Department of Statistics and Operations Research, University of Limpopo, Polokwane, Limpopo, South Africa; 2 Department of Mathematics and Statistics, Missouri University of Science and Technology, Rolla, Missouri, United States; Semnan University, IRAN, ISLAMIC REPUBLIC OF

## Abstract

In survival analysis, it is often assumed that all individuals will eventually experience the event of interest if followed long enough. However, in many real-world scenarios, a subset of individuals remains event-free indefinitely. For instance, in clinical studies, some patients never relapse and are considered cured rather than censored. Traditional survival models are inadequate for capturing this heterogeneity. Mixture cure models address this limitation by distinguishing between cured and susceptible individuals while modeling the survival of the latter. A key challenge in mixture cure modeling is selecting relevant covariates, particularly when dealing with time-varying effects. This study develops a penalized logistic/Cox proportional hazards mixture cure model incorporating time-varying covariates for both the incidence and latency components. The model is implemented using the smoothly clipped absolute deviation (SCAD) penalty to facilitate variable selection and improve model interpretability. To achieve this, we modified the penPHcure package to accommodate SCAD regularization and generate time-varying covariates. The proposed approach is applied to real-world data on the time to death for hospitalized COVID-19 patients in Limpopo Province, South Africa, demonstrating its practical applicability in survival analysis.

## 1 Introduction

A common assumption in survival analysis is that all study subjects will eventually encounter the event of interest if observed for a sufficient duration. However, that is not always the case. In practice, it is common for a portion of the subjects to never encounter the event of interest, even after an extensive period of follow-up. In clinical trials, for example, there exists a proportion of subjects who will never experience a relapse. These individuals are not censored in the conventional sense, instead, they are considered cured or immune. Traditional survival models are not suitable for analysing such cured subjects. Survival models that consider individuals who never experience an event are commonly known as cure models.

Choosing correct covariates for modelling to better understand an event has been a major problem for researchers. Variable selection is obviously not a simple task when dealing with the complicated structures of cure rate models [1–3]. There are many different ways of selecting variables. While most statisticians are familiar with methods of choosing variables, few are aware that such methods could lead to models with poor performance [4]. The study of [5] reviewed some popular variable selection algorithms. These algorithms include backward elimination; forward selection; stepwise forward selection; stepwise backward elimination; augmented backward elimination; best subset selection; least absolute shrinkage and selection operators (LASSO); and univariate selection [5].

The prediction accuracy of the regression model forms a key feature when evaluating a variable selection method [6]. This implies that the variables selected must provide the best fit for the model, ultimately yielding accurate predictions [7]. Other important requirements for a preferred variable selection method in a regression model are: interpretability, stability, parsimony, and bias avoidance when drawing inferences [4,5,7].

Regularisation is a technique that is almost always beneficial to the model’s prediction performance [8,9]. Regularisations are approaches for reducing error and avoiding over-fitting by suitably fitting a function on the supplied training set [10]. This technique prevents the model from over-fitting by adding a penalty. Various penalisation methods have been proposed and widely used during the past decade for variable selection in survival analysis. However, only a few studies focused on the penalised mixture cure models (MCMs) when a cure fraction exists.

Research by [11] examined variable selection techniques for the semi-parametric proportional hazards MCM, considering both LASSO and the smoothly clipped absolute deviation (SCAD) penalties. Another approach by [12] utilised a parametric MCM with an accelerated failure time (AFT) regression model for survival and extended generalised gamma distribution for the error term. Adaptive LASSO was also incorporated into this model. Additionally, [3] introduced two methods based on the LASSO for variable selection in both the MCM and the promotion time cure model, thus accommodating parametric or non-parametric baseline hazards.

A study by [13] extended the proportional hazards (PH) MCM to accommodate time-varying covariates, utilising the SCAD penalty. Another approach by [14] introduced a penalisation method for estimating the MCM, explicitly considering the structural effects of covariates. Their method provided more informative estimates compared to standard techniques and exhibited greater flexibility than existing approaches regarding structural effects. Depending on the data characteristics, they developed various penalties and corresponding computational algorithms. Furthermore, [15] devised a variable selection method for the semi-parametric non-mixture or promotion time cure model in the presence of interval-censored data with a cured subgroup. They proposed a penalised likelihood approach with the use of the LASSO, adaptive LASSO, or SCAD, which was easily accomplished via a novel penalised expectation-maximisation (EM) algorithm.

The penPHcure R package was introduced by [16], implementing the semi-parametric PH cure model and utilising variable selection techniques based on SCAD-penalised likelihood. On the other hand, [17] investigated variable selection in the context of interval-censored failure time data originating from a broad class of generalised odds rate MCMs. They proposed a penalised variable selection method by maximising a derived penalised likelihood function. This method employed the sieve approach to approximate the unknown function and was implemented using a novel penalised EM algorithm.

A study was conducted with the objective of devising penalised parametric MCMs suitable for high-dimensional datasets [18]. Their goal was to identify prognostic factors linked to both cure status and survival among susceptible individuals. For model estimation, they employed two distinct iterative algorithms: the generalised monotone incremental forward stagewise and the EM algorithm. In addition, these algorithms were integrated with the model-X knockoffs framework, which offers a flexible selection approach enabling strict control over the false discovery rate.

A method for accounting for non-linear influences and determining the composition of the cure rate model was suggested [19]. They specifically employed LASSO to choose linear and non-linear components after partitioning each variable into linear and non-linear components. They used cubic B-splines to operationally model the non-linear components. They employed the EM technique to determine the maximum likelihood estimations.

For the PH cure model, [20] presented a stepwise variable selection method with a logistic regression for the cure rate and a Cox regression for the hazards for patients who are not cured. They performed simulation studies to compare the performance of the stepwise method to that of the convenience variable selection method, which includes all variables in the PH cure model and only chooses the important variables, and the best subset selection method based on Akaike information criterion.

Using the Cox PH MCM and bounded cumulative hazard model, several estimators were proposed by [21]. These estimators utilise pseudo-observations to evaluate the impacts of covariates on both the cure rate and the risk of experiencing the event of interest in survival data with a cure fraction. Furthermore, a variable selection procedure based on the pseudo-observations was introduced, employing penalised generalised estimating equations for both the PH MCM and the bounded cumulative hazard models.

This work aims to develop a penalised logistic/Cox PH MCM that will be able to select variables that are truly associated with both cure status and survival of uncured subjects via an elastic net (EN) penalty. Traditional cure rate models operate under the assumption that the population comprises both cured and non-cured individuals. On the other hand, MCMs consider the population to be heterogeneous, comprising a mixture of cured individuals who will never experience events and susceptible individuals who will be subject to events. One advantage of the MCMs is that it distinguishes between modelling of the proportion of cured patients and the survival distribution of uncured patients, thus allowing for straightforward interpretation of the parameters associated with covariates **x** and **z** in the model.

The logistic model possesses several significant attributes. Notably, it is easy to interpret and estimate, and it is readily available in various statistical software packages. On the other hand, the Cox PH model, introduced by [22], is a regression model commonly employed in medical research to explore the relationship between patients’ survival time and one or more predictor variables. A key factor contributing to the widespread adoption of the Cox PH model is its ability to provide reasonably accurate estimates of regression coefficients, hazard ratios, and adjusted survival curves across a broad spectrum of data scenarios, even without specifying the baseline hazard.

The Cox PH model is considered robust, as its results closely approximate those of the correct parametric model. For instance, if the true parametric model is Weibull, employing the Cox PH model typically yields results comparable to those obtained with a Weibull model. Therefore, in situations of uncertainty, opting for the Cox PH model is regarded as a “safe” choice [23–25].

The EN penalty, introduced by [9], will be applied to the MCM. This penalty method is a form of regularised regression that combines the penalties of the LASSO and ridge methods. Similar to the LASSO, the EN method performs automatic variable selection and continuous shrinkage. Moreover, it also addresses the limitations of the LASSO by simultaneously incorporating both penalties.

The LASSO has certain limitations, especially in scenarios where the number of predictors (*p*) is large and the number of samples (*n*) is small, known as high-dimensional data with few examples. In such cases, the LASSO tends to select a maximum of *n* variables before reaching saturation. Additionally, when dealing with a group of highly correlated variables, it often selects only one variable from the group while ignoring the rest. The EN method addresses these limitations by acting like a stretchable fishing net that retains all significant variables. Simulation studies and real-world examples demonstrate that the EN method frequently outperforms the LASSO in terms of prediction accuracy [9].

## 2 Model and estimation

### 2.1 Model

The variable of interest is a non-negative random variable *T*, representing the time until the occurrence of the event of interest. We presume this variable is subject to random right censoring. Instead of directly observing *T*, we observe $t_{i}=\min(T_{i},C_{i})$ and $\delta_{i}=I(T_{i}\leq C_{i})$, where *I* ( ⋅ )  denotes the indicator function, and $C_{i}$ signifies the random censoring time. In the presence of a cure fraction, the survival function $\bar{F}(t)=P(T>t)$ of *T* is such that $\underset{t\to \infty }{\lim}\bar{F}(t)>0$. This limiting value, denoted by 1–*p*, represents the proportion of cured subjects, termed the cure rate.

Because of right censoring, $T_{i}$ is never directly observed once it reaches infinity. Specifically, when $\delta_{i}=1$ (uncensored observation), we can confidently determine that the individual is susceptible (uncured). However, in the case of $\delta_{i}=0$ (censored observation), the individual could belong to either of the subpopulation, but we lack the information to distinguish between them. It is commonly assumed that *T* and *C* are independent given the covariates ${\left(x^{\prime },z^{\prime }\right)}^{\prime }$.

Let $Y_{i}$ denote a variable indicating whether an individual is susceptible $\left(Y_{i}=1\right)$ or non-susceptible $\left(Y_{i}=0\right)$ to the event of interest, and let $T_{i}$ be a non-negative random variable representing the failure time of interest, defined only when $Y_{i}=1$. Consider $\left(t_{i},\delta_{i},x_{i},z_{i}\right)$, *i* = 1 , … , *n*, as *i* . *i* . *d* .  realisations of  ( *t* , *δ* , *x* , *z* ) . The survival function for the entire population is given by

$$\begin{array}{ccc} {\bar{F}}_{\text{pop}}\left(t|x_{i},z_{i}\right)=1-p\left(x_{i}\right)+p\left(x_{i}\right){\bar{F}}_{u}\left(t|z_{i}\right), & & \end{array}$$ (1)

where $p(x_{i})=P(Y_{i}=1|x_{i})$ represents the probability of susceptibility (commonly referred to as incidence), while ${\bar{F}}_{u}(t|Y_{i}=1,z_{i})=P(T>t|z_{i},Y_{i}=1)$ denotes the survival function for susceptible individuals (often termed latency). The logistic model is used to model the cure rate in general. The logistic incidence model for the probability of being uncured, along with the vector of time-varying covariates and the corresponding parameter vector *β* containing an intercept, can be expressed by:

$$\begin{array}{ccc} P\left(Y_{i}=1|x_{i}\left(t\right)\right)=p\left(x_{i}\left(t\right)\right)=\frac{\exp\left(\beta^{\prime }x_{i}\left(t\right)\right)}{1+\exp\left(\beta^{\prime }x_{i}\left(t\right)\right)}, & & \end{array}$$ (2)

where $x_{i}(t)$ represents a vector of time-varying covariates (including the intercept), and *β* is a vector of regression coefficients. For the latency, the conditional survival function is modelled using a Cox PH model. In the Cox PH model, let $z_{ij}(t)$ denote the $j^{th}$ covariate of the $i^{th}$ unit under observation, where *i* = 1 , … , *n*, *j* = 1 , … , *p*, and *t* is an observed value of the time scale. The notation $z_{ij}(t)$ signifies that the value of $z_{ij}$ varies as a function of the time scale. The survival function can be written as:

$$\begin{array}{ccc} \begin{array}{ccc} {\bar{F}}_{u}\left(t|Y_{i}=1,z_{i}\right) & ={\bar{F}}_{0}{\left(t|Y_{i}=1\right)}^{exp\left(\gamma^{\prime }z_{i}\right)} & \\ & =\exp\left(-\exp\left(\gamma^{\prime }z_{i}\right)\int_{0}^{t_{i}}\lambda_{0}\left(t|Y_{i}=1\right)du\right), \end{array} & & \end{array}$$ (3)

where ${\bar{F}}_{0}\left(t|Y_{i}=1\right)$ and $\lambda_{0}\left(t|Y_{i}=1\right)$ are the baseline conditional survival and hazard functions respectively. The conditional cumulative hazard function is $\Lambda \left(t|Y_{i}=1\right)=\Lambda_{0}\left(t|Y_{i}=1\right)\exp\left(\gamma^{\prime }z_{i}\right)$, where $\Lambda_{0}\left(t|Y_{i}=1\right)=\int_{0}^{t_{i}}\lambda_{0}\left(u|Y_{i}=1\right)du$. The hazard function corresponding to the survival function is then

$$\begin{array}{ccc} \lambda_{i}\left(t;z_{i}\left(t\right)\right)=\lambda_{0}\left(t\right)\exp\left(z_{i}\left(t\right)\gamma \right), & & \end{array}$$ (4)

where $\lambda_{0}$ represents the baseline hazard function, $z_{i}(t)$ is a 1 × *p* vector of time-dependent covariates for unit *i*, and *γ* is a *p* × 1 vector of coefficients.

### 2.2 Variable selection and estimation

Individual *i* in the dataset is represented by the observed data $\left(t_{i},\delta_{i},z_{i}\right)$, with $t_{i}$ denoting the observed event or censoring time. The indicator $\delta_{i}$ takes the value 1 if $t_{i}$ is uncensored and 0 otherwise. Additionally, $x_{i}=\left(1,{z_{i}}^{\prime }\right)$, although the covariates in $x_{i}$ and $z_{i}$ need not be identical. The *k* distinct event times are denoted by $t_{\left(1\right)},\ldots ,t_{\left(k\right)}$. If $\delta_{i}=1$, $y_{i}=1$, and if $\delta_{i}=0$, $y_{i}$ remains unobserved, where $y_{i}$ represents the value taken by the random variable $Y_{i}$. The likelihood contribution of individual *i* is $p_{i}f\left(t_{i}|Y=1;z_{i}\right)$ for $\delta_{i}=1$ and $\left(1-p_{i}\right)\;+\;p_{i}\bar{F}\left(t_{i}|Y_{i}=1;z_{i}\right)$ for $\delta_{i}=0$, where $p_{i}=Pr\left(Y_{i}=1;x_{i}\right)$. For the proportional hazards cure model, the observed full likelihood is then:

$$\begin{array}{ccc} \begin{array}{ccc} L\left(\beta ,\gamma ,\lambda_{0};y\right) & =\overset{n}{\underset{i=1}{\prod }}{\left\{p\left(x_{i}\right)\lambda_{0}\left(t_{i}|Y=1\right)e^{{z_{i}}^{\prime }\beta }e^{-\Lambda_{0}\left(t_{i}|Y=1\right)\exp\left({z_{i}}^{\prime }\beta \right)}\right\}}^{\delta_{i}} & \\ & \times {\left\{\left(1-p\left(x_{i}\right)\right)+p\left(x_{i}\right)e^{-\Lambda_{0}\left(t_{i}|Y=1\right)\exp\left({z_{i}}^{\prime }\right)\beta }\right\}}^{1-\delta_{i}}. \end{array} & & \end{array}$$ (5)

Estimation of the non-parametric baseline hazard involves maximising the likelihood in Eq 5. We employ an EM algorithm to maximise the complete likelihood based on $\left(t_{i},\delta_{i},x_{i},z_{i};y_{i}\right)$, *i* = 1 , *…* , *n*, while treating $y_{i}^{s}$ as a latent binary variable. The complete likelihood (Eq 6) comprises a logistic component for the cured individuals and a PH component for the non-cured individuals, and is given by:

$$\begin{array}{ccc} \begin{array}{cc} L\left(\beta ,\gamma ,\lambda_{0};y\right) & =\overset{n}{\underset{i=1}{\prod }}\left[p{\left(x_{i}\right)}^{y_{i}}1-p{\left(x_{i}\right)}^{1-y_{i}}\right]\overset{n}{\underset{i=1}{\prod }}{\left[{\left\{\lambda_{0}\left(t_{i}\right)\exp\left(\gamma^{\prime }z_{i}\right)\right\}}^{\delta_{i}}{\bar{F}}_{0}{\left(t_{i}\right)}^{e^{\prime }z_{i}}\right]}^{y_{i}}. \end{array} & & \end{array}$$ (6)

The log-likelihood function can be expressed as the sum of two components, each pertaining solely to the incidence component or latency component:

$$\begin{array}{ccc} \begin{array}{ccc} l\left(\beta ,\gamma ,\lambda_{0};y\right) & =\overset{n}{\underset{i=1}{\sum }}\left\{y_{i}\beta^{\prime }x_{i}-\log\left\{1+\exp\left(\beta^{\prime }x_{i}\right)\right\}\right\} & \\ & +\overset{n}{\underset{i=1}{\sum }}\left\{y_{i}\delta_{i}\left\{\log\left\{\lambda_{0}\left(t_{i}\right)\right\}+\gamma^{\prime }z_{i}\right\}+y_{i}\exp\left(\gamma^{\prime }z_{i}\right)\log\left\{\bar{F}\left(t_{i}\right)\right\}\right\}. \end{array} & & \end{array}$$ (7)

For simplicity, we write the first term of Eq 7 as $l_{1}\left(\beta ;y\right)$ and the second term as $l_{2}\left(\gamma ,\lambda_{0};y\right)$. To achieve sparse estimation, we apply the EN method, which imposes penalties in the form of $L_{1}$ and $L_{2}$ norms on the log likelihood:

$$\begin{array}{ccc} \begin{array}{ccc} l_{p}\left(\beta ,\gamma ,\lambda_{0};y\right) & =\left\{l_{1}\left(\beta ;y\right)+\lambda_{2}\overset{k}{\underset{j=1}{\sum }}\beta_{j}^{2}+\lambda_{1}\overset{k}{\underset{j=1}{\sum }}|\beta_{j}|\right\} & \\ & +\left\{l_{2}\left(\gamma ,\lambda_{0};y\right)+\lambda_{2}\overset{k}{\underset{j=1}{\sum }}\gamma_{j}^{2}+\lambda_{1}\overset{k}{\underset{j=1}{\sum }}|\gamma_{j}|\right\}, \end{array} & & \end{array}$$ (8)

where $\lambda_{1}$ and $\lambda_{2}$ denote the amount of shrinkage or tuning parameters controlling the amount of penalty. The naive EN estimators $\hat{\beta }$ and $\hat{\gamma }$ are the minimisers of Eq 8:

β^=arg ⁡ min ⁡ β {L (λ1,λ2,β)}, (9)

and

γ^=arg ⁡ min ⁡ γ {L (λ1,λ2,γ)}. (10)

This approach can be interpreted as a penalised least squares technique. If we define $\alpha =\lambda_{2}∕\left(\lambda_{1}+\lambda_{2}\right)$, solving for $\hat{\beta }$ and $\hat{\gamma }$ in Eq 8 is equivalent to solving the optimisation problem. The terms $\left(1-\alpha \right)|\beta {|}_{1}\;+\;\alpha |\beta {|}^{2}$ and $\left(1-\alpha \right)|\gamma {|}_{1}\;+\;\alpha |\gamma {|}^{2}$ are known as the EN penalty, representing a convex combination of the LASSO and ridge penalties. When *α* = 1, the naive EN simplifies to the ridge regression. In this study, we focus on the case where *α* < 1. For all *α* ∈ [ 0 , 1 ] , the EN penalty function is singular (i.e., the first derivative does not exist) at 0, but remains strictly convex for all *α* > 0, thereby combining features of both the LASSO and the ridge regression. It is worth noting that the LASSO penalty  ( *α* = 0 )  is convex but not strictly convex. Consequently, the log likelihood function is given by:

$$\begin{array}{ccc} \begin{array}{ccc} l_{p}\left(\beta ,\gamma ,\lambda_{0};y\right) & =\left\{l_{1}\left(\beta ;y\right)+\lambda_{\beta }\left(1-\alpha \right)|\beta {|}_{1}+\alpha |\beta {|}^{2}\right\} & \\ & +\left\{l_{2}\left(\gamma ,\lambda_{0};y\right)+\lambda_{\gamma }\left(1-\alpha \right)|\gamma {|}_{1}+\alpha |\gamma {|}^{2}\right\}, \end{array} & & \end{array}$$ (11)

where $\lambda_{\beta }$ and $\lambda_{\gamma }$ are tuning parameters for $\beta^{\prime }s$ and $\gamma^{\prime }s$ respectively.

**Computation**: For computation, we use EN estimates $\left(\hat{\beta },\hat{\gamma }\right)$ and the quadratic approximation algorithm. Since $y_{i}=1$ when $\delta_{i}=1$, but remains unobserved when $\delta_{i}=0$, we estimate the unknown parameter $\theta =\left(\gamma ,\beta ,\lambda_{0}\right)$ using the EM algorithm. With initial values $\theta^{\left(0\right)}$, the $m^{th}$ iteration of EM comprises two steps:

**E-step:** The E-step in the ${\left(m+1\right)}^{th}$ iteration is used to calculate the expected $l_{p}\left(\theta \right)$ with respect to the conditional distribution of $Y_{i}$ given the current parameter estimates $\hat{\theta }$ and the observed data $O_{i}$ at the $m^{th}$ iteration. Since the $Y_{i}$‘s are linear terms l (θ), we only need to compute the expected value of $Y_{i}$ given $\hat{\theta }$ and $O_{i}$, denoted by $\hat{p}=E\left(Y_{i}|\hat{\theta },O_{i}\right)=Pr\left(Y_{i}=1|\hat{\theta },O_{i}\right)$.

When subject *i* is censored with $\delta_{i}=0$, we have

$$\begin{array}{ccc} {\hat{p}}_{i}=\frac{p{\left(x_{i}\right)}^{m}{\bar{F}}_{0}^{m}{\left(t_{i}\right)}^{e^{\gamma^{\left(m\right)\prime }z_{i}}}}{1-p{\left(x_{i}\right)}^{m}\left\{1-{\bar{F}}_{0}^{m}{\left(t_{i}\right)}^{e^{\gamma^{\left(m\right)\prime }z_{i}}}\right\}}. & & \end{array}$$ (12)

When an event is observed for subject *i* with $\delta_{i}=1$, ${\hat{p}}_{i}$ is equal to 1.

The binary indicator variable is estimated as follows: Estimate of $P\left(Y_{i}=1\right)=\hat{p}$

$$\begin{array}{ccc} P\left(Y_{i}=1\right) & =P\left(T\leq t_{i}|\delta_{i}=1\right)\cdot \left(\delta_{i}=1\right)+P\left(T\leq t_{i}|\delta_{i}=0\right)\cdot P\left(\delta_{i}=0\right) & \\ & =1-\bar{F}\left(t_{i}|\delta_{i}=1\right)\\ & =1\left(1-P\left(T\geq t_{i}|\delta_{i}=1\right)\right) \end{array}$$

$$\begin{array}{ccc} \bar{F}\left(t_{i}|\delta_{i}=1\right) & =p\left(x_{i}\right)+\left(1-p\left(x_{i}\right)\right)P\left(T\geq t_{i}|\delta_{i}=1\right) & \\ & =P\left(Y_{i}=1|z_{i}\right)\cdot P\left(T\geq t_{i}|z_{i}\right)\\ & =1-P\left(Y_{i}=1|z_{i}\right)+P\left(Y_{i}=1|z_{i}\right)\cdot P\left(T>t_{i}|z_{i}\right) \end{array}$$

$$\bar{F}\left(t_{i}|\delta_{i}=1\right)=p\left(x_{i}\right)+\left(1-p\left(x_{i}\right)\right)\times \delta_{i}+\left(1-\delta_{i}\right)$$

$${\hat{p}}_{i}^{m}=\delta_{i}+\left(1-\delta_{i}\right)\frac{p{\left(x_{i}\right)}^{\left(m\right)}{\bar{F}}_{0}{\left(t_{i}\right)}^{e^{\gamma {\left(m\right)}^{\prime }z_{i}}}}{1-p{\left(x_{i}\right)}^{\left(m\right)}\left\{1-{\bar{F}}_{0}^{{\left(m\right)}^{\prime }z_{i}}\right\}}.$$

To obtain the expected l (θ), the E-step replaces $y_{i}$ in the log likelihood with ${\hat{p}}_{i}$.

**M-step:** With ${\hat{p}}_{i}$ plugged in, we maximise the penalised log likelihood $l_{p}$ with respect to $\beta ,\gamma ,\lambda_{0}$. The M-step involves the following sub-steps:

- Estimate the cumulative hazard function $\Lambda_{0}\left(t\right)$ using the Breslow estimator [26]. Using $l_{2}\left(\gamma ,\lambda_{0};y\right)$, it can be shown that the non-parametic maximum likelihood estimator of $\Lambda_{0}\left(t|Y=1\right)$ given *γ* is a slight modification of the Nelson-Aalen estimator. The Nelson-Aalen estimator is given by [27]:
  $$\begin{array}{ccc} \hat{\Lambda }\left(t\right)=\underset{t_{i}\leq t}{\sum }\frac{d_{i}}{n_{i}}, & & \end{array}$$ (13)
  where $d_{i}$ is the number of events at $t_{i}$ and $n_{i}$ is the total number of individuals at risk at $t_{i}$. Breslow estimator was proposed as an improvement of the Nelson-Aalen to include covariates. The Cox PH model, introduced by [22], is a regression model that specifies the conditional hazard function of the failure time for a given set of covariates. The hazard function is then defined by
  $$\begin{array}{ccc} \lambda \left(t|z_{i}\right)=\lambda_{0}\left(t\right)\exp\left(\gamma^{\prime }z_{i}\right), & & \end{array}$$ (14)
  where $z={\left(z_{1},...,z_{p}\right)}^{\prime }$ is a *p* − dimensional vector of covariates, $\gamma ={\left(\gamma_{1},...,\gamma_{p}\right)}^{\prime }$ is a vector of regression coefficients and $\lambda_{0}\left(t\right)$ is the baseline hazard function. Consider a set of *n* independent subjects such that the counting process $\left\{N_{i}\left(t\right);t\geq 0\right\}$ for the ith subject in the set records the number of observed events up to time *t*. The intensity function for $N_{i}\left(t\right)$ is given by
  $$\begin{array}{ccc} Y_{i}\left(t\right)d\Lambda \left(t;z_{i}\right)=Y_{i}\left(t\right)e^{\gamma^{\prime }z_{i}\left(t\right)}d\Lambda_{0}\left(t\right), & & \end{array}$$ (15)
  where $Y_{i}\left(t\right)$ is a predictable process that can take values in the set  {0,1}. When it takes the value 1, it indicates that the *i^th^* individual is at risk at time $t_{i}$. Additionally, $z_{i}\left(\cdot \right)$ is the covariate process for the $i^{th}$ subject.
  The cumulative hazard function is defined as [26]:
  $$\begin{array}{ccc} \Lambda_{0}\left(t\right)=\int_{0}^{t}\lambda_{0}\left(u\right)du. & & \end{array}$$ (16)
  The counting process $N_{i}\left(\cdot \right)$ can be uniquely decomposed for every *i* and *t*,
  $$\begin{array}{ccc} N_{i}\left(t\right)=M_{i}\left(t\right)+\int_{0}^{t}Y_{i}\left(u\right)d\Lambda \left(u;z_{i}\right), & & \end{array}$$ (17)
  where $M_{i}\left(\cdot \right)$ is a local square integrable martingale. In view of the relationship in Eq 13, it is natural to estimate $\Lambda_{0}\left(t\right)$ by
  $$\begin{array}{ccc} \tilde{\Lambda_{0}}\left(\hat{\gamma },t\right)=\int_{0}^{t}\frac{\overset{n}{\underset{i=1}{\sum }}dN_{i}\left(u\right)}{\overset{n}{\underset{j=1}{\sum }}Y_{i}\left(u\right)e^{\hat{\gamma }z_{i}\left(u\right)}}, & & \end{array}$$ (18)
  where $\hat{\gamma }$ is a consistent estimator.
- Solve the penalised score equation for the $\beta^{\left(m+1\right)}$ in the logistic model
  $$\begin{array}{ccc} 0=U\left(\beta \right)=\overset{n}{\underset{i=1}{\sum }}\left[{\hat{p}}_{i}-\frac{\exp\left(\beta^{\prime }x_{i}\right)}{1+\exp\left(\beta^{\prime }x_{i}\right)}\right]x_{i}^{\prime }+\lambda_{\beta }\left(1-\alpha \right)|\beta {|}_{1}+\alpha |\beta {|}^{2}|. & & \end{array}$$ (19)
  We obtained the penalty term $\lambda_{\beta }\left(1-\alpha \right)|\beta {|}_{1}+\alpha |\beta {|}^{2}$ by using a quadratic approximation of the penalised likelihood. The penalised Hessian matrix $H_{\beta }$ for *β* is given by $H_{\beta }={△}_{\beta }U_{\beta }$.
- Solve the penalised score equation for the survival model with respect to $\gamma^{\left(m+1\right)}$ given $\Lambda_{0}^{\left(m+1\right)}\left(t\right)$, $\beta^{\left(m+1\right)}$
  $$\begin{array}{ccc} \begin{array}{ccc} 0=U\left(\gamma \right) & =\overset{n}{\underset{i=1}{\sum }}\left[{\hat{p}}_{i}\delta_{i}-{\hat{p}}_{i}\exp\left(\gamma^{\prime }z_{i}\right)\Lambda_{0}^{\left(m+1\right)}\left(t_{i}\right)\right]{z_{i}}^{\prime } & \\ & +\lambda_{\gamma }\left(1-\alpha \right)|\gamma {|}_{1}+\alpha |\gamma {|}^{2} \end{array} & & \end{array}$$ (20)
  We obtained the penalty terms $\lambda_{\gamma }\left(1-\alpha \right)|\gamma {|}_{1}+\alpha |\gamma {|}^{2}$ by using a quadratic approximation of the penalised likelihood. The penalised Hessian matrix $H_{\gamma }$ for *γ* in the ${\left(m+1\right)}^{th}$ iteration is given by $H_{\gamma }={△}_{\gamma }U_{\gamma }$.

The M-step iterates through the above sub-step until convergence is achieved. The final maximum likelihood estimates $\hat{\beta },\hat{\gamma }$ are achieved by iterating between the E and the M steps. The estimator $\hat{\gamma }$ is obtained by setting the likelihood equation to zero. This estimator has favourable asymptotic properties, as shown by various authors.

In summary, the key steps of the EM algorithms are:

**Step 1:** Fix the tuning parameters $\left(\lambda_{\beta },\lambda_{\gamma }\right)$ and initialise $\left(\beta^{\left(0\right)},\gamma^{\left(0\right)},\lambda_{0}^{\left(0\right)}\left(t\right)\right)$
**Step 2:** Execute the E-step and compute $\lambda_{0}\left(t\right)$
**Step 3:** Update the estimates as $\beta^{\left(1\right)}=\beta^{\left(0\right)-H^{-1}}\left(\beta^{\left(0\right)}\right)U\left(\beta^{\left(0\right)}\right)$ for the logistic regression and $\gamma^{\left(1\right)}=\gamma^{\left(0\right)}-H^{-1}\left(\gamma^{\left(0\right)}\right)U\left(\gamma^{\left(0\right)}\right)$ for the survival model
**Step 4:** Repeat steps 2 and 3 until $|\beta^{\left(1\right)}-\beta^{\left(0\right)}|\to 0$ and $|\gamma^{\left(1\right)}-\gamma^{\left(0\right)}|\to 0$.

**Tuning/regularisation parameters selection** Tuning parameter selection is vital in the optimisation of the penalised least squares estimators for achieving consistent selection and optimal estimation. To select the proper tuning parameter, the existing literature offers two frequently applied approaches, which are, the CV approach and the information criterion-based approach. Bayesian information criterion (BIC), generalised information criterion and Akaike information criterion (AIC) are some of the information criterion based approaches.

Choosing appropriate tuning parameters $\left(\lambda_{\beta },\lambda_{\gamma }\right)$ is essential for variable selection. There is a trade-off between bias and variance in resulting estimators. As *λ* increases, bias increases and as *λ* decreases, variance increases. As *λ* increases, more coefficients shrink to zero [3,28]. At the same time, estimates of non-zero coefficients are likely to have increased biases [3,29].

## 3 Simulation study

The first objective of the simulation study is to investigate the behaviour of the logistic/Cox PH cure model for time-varying covariates on both latency and incidence. Secondly, it aims to investigate the impact of the performance of cured right-censored observations on the study results. Furthermore, the study conducts an evaluation of the EN procedure, examining both its estimation capabilities and its effectiveness in selecting relevant variables.

### 3.1 Modification of penPHcure package

The penPHcure package is tailored for simulating time-invariant covariates for incidence and time-varying covariates for latency. Developed exclusively in R Studio, it is designed to support LASSO and SCAD penalties. However, this current research endeavours to enhance the versatility of the package by incorporating both the EN penalty and time-varying covariates for both incidence and latency. To achieve this objective, a meticulous and thorough editing process was undertaken on the penPHcure package, resulting in a locally customised version that has been nicknamed “PenPHcure.AaRN". The key modification we introduced was to add support for the EN penalty and extend the package’s ability to simulate time-varying covariates; not only for latency, but also for incidence. This enhancement required a detailed and thorough adding (editing) of the package’s internal code (on the R/ and src/ directories which are key components of the package structure), leading to the development of a customised version named “PenPHcure.AaRN". This modified package does not only provide the capacity to utilise the EN penalty, but also to generate time-varying covariates for both incidence and latency; seamlessly integrating these capabilities with its original functionalities.

### 3.2 Data generation

This simulation study follows the settings in penPHcure.simulate package within the R studio environment. For a comprehensive understanding of the data generation process, see [16]. The penPHcure package generates data with time-varying covariates for latency while maintaining the covariates for incidence as time-invariant. Notably, this study incorporates time-varying covariates for both latency and incidence in the data generation process.

To generate the data, PenPHcure.AaRN package, which is similar to penPHcure package by [16], was used. Let $S=\left\{s_{1},s_{2},...,s_{J}\right\}$ be a partition of the time scale forming *J* + 1 intervals $\left(0,s_{1}],(s_{1},s_{2}],\cdots \;,(s_{j-1},s_{J}],(s_{J},\infty \right]$. Generate time-varying covariate vectors that are piecewise constant for each interval for $\text{z}\left(t\right)={\text{z}}_{j}$ and $\text{x}\left(t\right)={\text{x}}_{j}$ for $t\in (s_{j-1},s_{j}]$. Consider a transformation *g* with the following properties: *g*(0) is set to 0, *g*(*t*) increases continuously as *t* becomes greater than 0, and the inverse of *g*, denoted as $g^{-1}(t)$, is smooth and differentiable. In the implementation of the penPHcure.simulate function, we utilise the transformation $g(t)=t^{1∕\gamma }$, where the parameter *γ* can be specified by the user via the argument gamma, and its default value is 1. According to [30], if we generate a random variable *V* as a piecewise exponential distribution with a density function given by:

$$\begin{array}{ccc} f_{V}(t)=\{\begin{array}{cc} \begin{array}{ccc} & \overset{j-1}{\underset{l=1}{\prod }}\exp\left\{-\lambda_{l}[g^{-1}(s_{l})-g^{-1}(s_{l-1})]\right\} & \\ & \times \lambda_{j}\exp\left\{-\lambda_{j}[t-g^{-1}(s_{j-1})]\right\}, \end{array}\; & \text{for }t\in (g^{-1}(s_{j-1}),g^{-1}(s_{j})]. \end{array} & & \end{array}$$ (21)

where $\lambda_{j}=\exp(z_{j}^{\prime }\beta )$ is the constant hazard in the interval $\left(g^{-1}(s_{j-1}),g^{-1}(s_{j})\right]$, then *g*(*V*) follows a Cox PH model with time-varying covariates, featuring a baseline hazard function given by $h_{0}(t)=\frac{d}{dt}[g^{-1}(t)]$. This method is part of the algorithm implemented in the penPHcure.simulate function for simulating data from a PH cure model with time-varying covariates. Table 1 shows a comprehensive explanation.

**Table 1:** Data generation algorithm.

Define *N* (sample size); *S* (Partition of the time scale); *β* (incidence coefficients); *γ* (latency coefficients).
**For** *i* = 1 , ⋯ , *N* **do**
**1:** Generate $x_{i}=\{x_{i,1},x_{i,2},\cdots \;,x_{i,J}\}$ from an arbitrary distribution;
**2:** Generate $y_{i}$ from a Bernoulli distribution with probability $p(x_{i})$;
**3:** Generate $z_{i}=\{z_{i,1},z_{i,2},\cdots \;,z_{i,J}\}$ from an arbitrary distribution;
**4:** Generate $v_{i}$ from a piecewise exponential distribution with density $f_{v}(t)$;
**5:** Compute $w_{i}=g(v_{i})$;
**6:** Generate $c_{i}$ from an arbitrary distribution;
**If** $y_{i}=0$ or $w_{i}>c_{i}$
$t_{i}=c_{i}$;
$\delta_{i}=0$;
**Else**
$t_{i}=w_{i}$;
$\delta_{i}=1$;
**End If**
**End For**
**Return** $\left\{(t_{i},\delta_{i},z_{i},x_{i});i=1,\ldots ,N\right\}$

### 3.3 Simulation setting

In this section, we present the results of an extensive simulation study meticulously designed to rigorously assess the finite sample performance of both the estimation of the PH cure model and its associated variable selection technique, thoughtfully implemented within the framework of the PenPHcure.AaRN function. These simulations are instrumental in shedding light on the practical effectiveness and robustness of our modelling approach.

To replicate real-world scenarios, the event times in our study were intentionally generated to conform to the Cox PH model; a fundamental and widely-used statistical model in survival analysis. Specifically, the baseline hazard function $h_{0}(t)$ was designed as a polynomial of the form $3t^{2}$, thereby introducing a non-linear component. This choice allowed us to evaluate the model’s ability to capture complex dependencies. Furthermore, our simulations involved the inclusion of 8 time-varying covariates, each contributing to the multifaceted nature of the data.

By undertaking this simulation study, we aim to provide a comprehensive empirical evaluation of the proposed methodology, thus offering insights into its practical utility and limitations. These covariates are assumed to remain constant within a set of 30 equally spaced intervals, outlined as $\left(0,s_{1}\right]$, $\left(s_{1},s_{2}\right]$,  … , $\left(s_{J-1},s_{J}\right]$, where the interval boundaries are defined as $s_{1}=0.2$ and $s_{J}=6$. This segmentation of the time scale into 30 intervals allows us to examine the behaviour of the covariates across different time segments with a granularity defined by these intervals.

The cure indicators are generated through a logistic regression model that includes 8 time-varying covariates, represented by the vector $x={\left(x_{1},\cdots \;,x_{8}\right)}^{\prime }$. These covariates are assumed to follow a multivariate normal distribution, denoted by ${\text{x}}_{ij}∼N\left(0,\sum \right)$ where $\sum_{p,q}=0.5^{\left|p-q\right|}$, for *p* , *q* = 1 , ⋯ , 8. In the logistic regression component of the mixture cure model, the regression coefficients are defined by the vector $\beta ={\left(b_{0},1.5,0,-0.75,0,-1.5,0,0.75,0\right)}^{\prime }$, where, $b_{0}$ represents the intercept term. In the logistic regression model, we include an intercept term, whereas in the survival model, we do not include an intercept. For the survival model, we also consider a scenario where $z={\left(z_{1},\cdots \;,x_{8}\right)}^{\prime }$ has 8 time-varying covariates that follow a multivariate normal distribution ${\text{z}}_{ij}∼N\left(0,\sum \right)$ where, $\sum_{p,q}=0.5^{\left|p-q\right|}$, for *p* , *q* = 1 , ⋯ , 8. In the latency component of the mixture cure model, the true coefficients are set to be $\beta_{0}={\left(-0.7,0,1,0,-0.5,0.75,0,0\right)}^{\prime }$. The failure times are generated from a Weibull distribution truncated at time 6, and any value greater than 6 will be censored.

In our analysis, we investigate six (6) simulation scenarios, each distinguished by different levels of censoring and proportions of cured individuals. These proportions are expressed as fractions of the sample size and are determined based on specific values for each scenario. Our analysis considers three different sample sizes, namely, N= {250,500,1000}. For each setting, we generated 500 replications using a dataset we created that includes time-varying covariates for both latency and incidence. Replicating 500 times provides a balance between computational efficiency and ensuring reliable, accurate estimates in the simulation results. Table 2 presents the various settings simulated in this study.

**Table 2:** Simulation settings.

Sample size	Censoring	Cure	$b_{0}$	$\lambda_{C}$
*N* = 250 , 500 , 1000	Low (40%)	High (30%)	1.45	0.02
*N* = 250 , 500 , 1000	Low (30%)	Medium (20%)	2.45	0.1
*N* = 250 , 500 , 1000	Medium (50%)	High (30%)	1.40	0.4
*N* = 250 , 500 , 1000	Medium (60%)	Medium (30%)	1.5	0.95
*N* = 250 , 500 , 1000	High (70%)	High (60%)	0.5	0.9
*N* = 250 , 500 , 1000	High (70%)	Medium (30%)	–0.7	0.4

In our study, we meticulously designed a tuning grid for the EN regularisation technique within the context of the MCM. This grid is composed of various combinations of hyperparameters, thus allowing us to thoroughly explore the model’s performance across a range of settings. The tune grid is defined as follows:

$$\begin{array}{ccc} \begin{array}{ccc} & \text{Pen.tuneGridEN}=\text{<- list(} & \\ & \text{CURE = list(lambda = exp(seq(-7, -2, length.out = 10))},\\ & \text{alpha = seq(0.1, 0.9, length.out = 5))}\\ & \text{SURV = list(lambda = exp(seq(-7, -2, length.out = 10))},\\ & \text{alpha = seq(0.1, 0.9, length.out = 5)))} \end{array} & & \end{array}$$ (22)

For the cure component, we specified a list of lambda values obtained by exponentiating a sequence spanning from -7 to -2, encompassing 10 equidistant points. Additionally, we incorporated alpha values within the range of 0.1 to 0.9, equally divided into 5 points. This comprehensive grid for the cure component empowers us to systematically evaluate how different combinations of lambda and alpha impact the model’s performance. Similarly, for the survival component, we employed a similar approach, constructing a tuning grid with lambda values ranging from -7 to -2, divided into 10 points, and alpha values distributed from 0.1 to 0.9 across 5 points.

This meticulous grid construction allows us to conduct a comprehensive analysis of the performance of the EN method and its adaptability to various hyperparameter settings, thus contributing to a more nuanced understanding of its efficacy within the MCM framework.

For all simulated datasets, we utilise the PenPHcure.AaRN function to perform the following analyses:

(i) Fit a standard PH cure model with all covariates (**FULL**).
(ii) Fit a standard PH cure model with only the covariates associated with non-zero coefficients (**ORACLE**).
(iii) Perform variable selection using the regularisation method with **LASSO** penalties, with tuning parameters chosen based on the BIC.
(iv) Perform variable selection using the regularisation method with **ELASTIC NET** penalties, with tuning parameters chosen based on the BIC.

## 4 Simulations results

**Fig 1:**
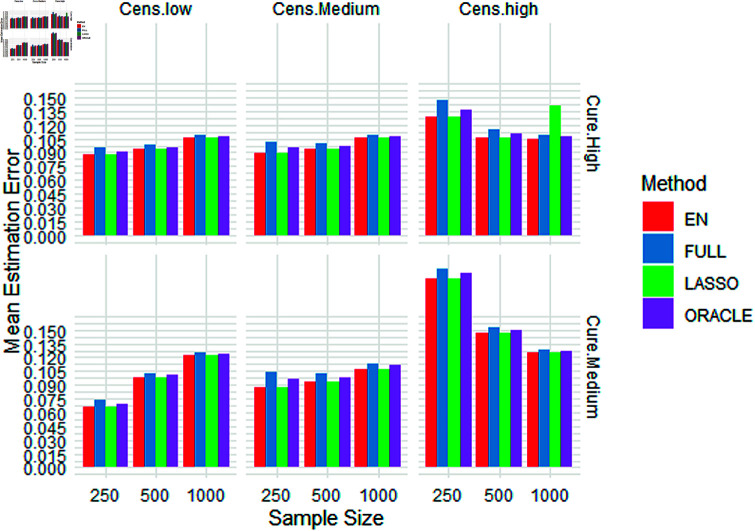
Simulation results: mean estimation errors (Incidence component).

**Fig 2:**
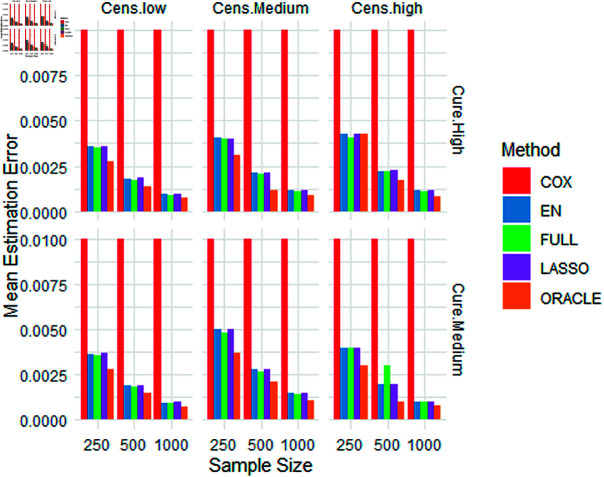
Simulation results: mean estimation errors (Latency component).

In Figures 1 and 2, we present visualisations of mean estimation errors (MEEs) for the latency and incidence components, respectively. These visualisations highlight how well our models capture the underlying patterns in each component.

In terms of the incidence component, we observe from Table 3 that the MEEs associated with the EN method appear to be almost equal to those of the LASSO method. However, it is noteworthy that the EN MEEs were found to be lower than the LASSO MEEs. Shifting our focus to the latency component, we again note that the MEEs produced by the EN approach seem to be equal on the graph to those originating from the LASSO method. Notably, EN MEEs are consistently lower compared to LASSO errors, as detailed in Table 4. This compelling evidence leads us to the conclusion that the adoption of EN within the framework of the MCM demonstrates superior performance compared to its LASSO counterpart.

**Fig 3:**
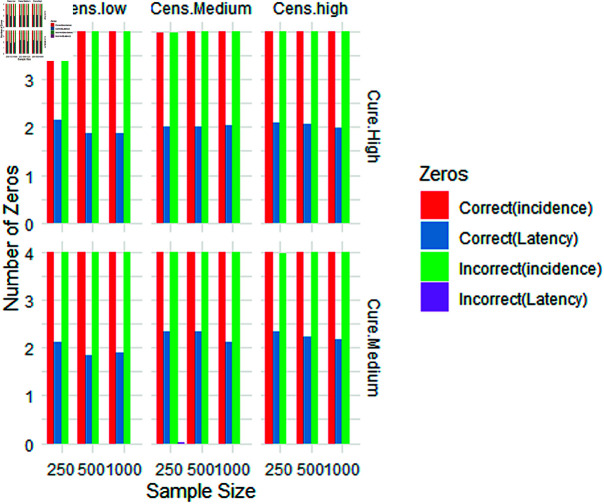
Simulation results: average number of correct/incorrect zeros identified by the variable selection technique (Elastic net penalty).

## 5 Simulations summary tables

Tables 3 and 4 present a comprehensive display of simulation results across various experimental scenarios. These tables systematically show different levels of censoring and proportions of cured individuals of the sample. These proportions are expressed as fractions of the sample size. In addition to these essential statistics, the tables report two key metrics, namely: the MEE and the mean relative estimation error (MREE). These metrics play a pivotal role in the assessment of model accuracy and predictive performance. Moreover, the tables record the average count of correctly estimated zeros (C-0’s) and incorrectly estimated zeros (IC-0’s), which are instrumental in understanding the effectiveness of the applied models. This comprehensive set of statistical data serves as a valuable tool for evaluating the performance of various models, with particular emphasis on the efficacy of the EN penalty model. The insights derived from these results contribute significantly to the assessment of model suitability and the determination of the superior model in survival analysis scenarios.

**Table 3:** Simulation results for scenarios with low censoring and low cure status.

				Incidence				Latency			
cens	cure	N	Method	MSE	MREE	C-0’s	IC-0’s	MSE	MREE	C-0’s	IC-0’s
0.4	0.3	250	FULL	0.094935	1	0	0	0.003472	1	0	0
			ORACLE	0.090867	0.956263	4	0	0.002750	0.795187	4	0
			LASSO	0.087297	0.952423	3.996	3.978	0.003565	1.055898	2.292	0
			EN	0.087249	0.951881	3.996	3.982	0.001801	1.056892	1.884	0
			COX	-	-	-	-	0.091649	58.197342	4	0
		500	FULL	0.098225	1	0	0	0.001737	1	0	0
			ORACLE	0.095833	0.975602	4	0	0.001380	0.796800	4	0
			LASSO	0.093721	0.970787	3.986	3.988	0.001815	1.068117	2.168	0
			EN	0.093682	0.970256	3.984	3.988	0.001801	1.056892	1.884	0
			COX	-	-	-	-	0.091649	58.197342	4	0
		1000	FULL	0.109080	1	0	0	0.000895	1	0	0
			ORACLE	0.107884	0.988971	4	0	0.000712	0.795965	4	0
			LASSO	0.106608	0.983982	4	3.996	0.000978	1.120992	2.244	0
			EN	0.106580	0.983712	4	3.994	0.000942	1.071858	1.882	0
			COX	-	-	-	-	0.120263	151.405197	4	0
0.3	0.2	250	FULL	0.072973	1	0	0	0.003586	1	0	0
			ORACLE	0.068986	0.942980	4	0	0.002807	0.784968	4	0
			LASSO	0.065881	0.936904	3.992	3.986	0.003668	1.050420	2.294	0
			EN	0.065908	0.937171	3.984	3.98	0.003645	1.042400	3.98	0
			COX	-	-	-	-	0.046748	14.508741	4	0
		500	FULL	0.102845	1	0	0	0.001849	1	0	0
			ORACLE	0.100461	0.976809	4	0	0.001460	0.976809	4	0
			LASSO	0.098616	0.960265	3.99	3.99	0.001928	1.076152	2.066	0
			EN	0.098601	0.968878	3.99	3.99	0.001910	1.059792	1.846	0
			COX	-	-	-	-	0.088670	53.462936	4	0
		1000	FULL	0.125188	1	0	0	0.000904	1	0	0
			ORACLE	0.123943	0.990003	4	0	0.000717	0.786186	4	0
			LASSO	0.122646	0.983745	3.992	3.994	0.000998	1.141715	2.27	0
			EN	0.122618	0.983502	3.996	3.994	0.000960	1.085519	1.896	0
			COX	-	-	-	-	0.115404	140.644574	4	0
0.5	0.3	250	FULL	0.101368	1	0	0	0.004004	1	0	0
			ORACLE	0.095910	0.945119	4	0	0.003101	0.778854	4	0
			LASSO	0.088934	0.914666	3.982	3.984	0.003997	1.022442	2.162	0
			EN	0.088917	0.914567	3.982	3.978	0.004023	1.032642	2.012	0
			COX	-	-	-	-	0.039050	10.872752	4	0
		500	FULL	0.099314	1	0	0	0.002116	1	0	0
			ORACLE	0.096182	0.968142	4	0	0.011613	0.779192	4	0
			LASSO	0.094309	0.973514	3.992	3.992	0.002125	1.059293	2.276	0
			EN	0.094308	0.973492	3.99	3.996	0.002116	1.051123	2.02	0
			COX	-	-	-	-	0.073079	39.117428	4	0
		1000	FULL	0.106210	1	0	0	0.001084	1	0	0
			ORACLE	0.108030	0.986271	4	0	0.000851	0.789375	4	0
			LASSO	0.001159	0.979479	3.996	3.994	0.001159	1.097099	2.264	0
			EN	0.106	0.979415	0	0	0.001144	1.082129	2.032	0
			COX	-	-	-	-	0.095950	98.137982	4	0

**Table 4:** Simulation results for scenarios with high censoring and high cure status.

				Incidence				Latency			
**cens**	**cure**	**N**	**Method**	**MEE**	**MREE**	**C-0’s**	**IC-0’s**	**yMEE**	**MREE**	**C-0’s**	**IC-0’s**
0.6	0.3	250	FULL	0.104581	1	0	0	0.004785	1	0	0
			ORACLE	0.095738	0.916011	4	0	0.003634	0.761960	4	0
			LASSO	0.087563	0.894843	3.974	3.976	0.004940	1.062800	2.468	0.006
			EN	0.087562	0.894914	3.978	3.97	0.004854	1.046466	2.322	0.004
			COX	-	-	-	-	0.026689	6.250201	4	0
		500	FULL	0.102915	1	0	0	0.002633	1	0	0
			ORACLE	0.984835	0.957014	4	0	0.002000	0.764368	4	0
			LASSO	0.093982	0.949700	3.99	3.988	0.002802	1.090088	2.458	0
			EN	0.094006	0.950222	3.982	3.984	0.002738	1.060672	2.458	0
			COX	-	-	-	-	0.047643	20.183577	4	0
		1000	FULL	0.113220	1	0	1	0.001402	1	0	0
			ORACLE	0.110884	0.979188	4	0	0.001077	0.771711	4	0
			LASSO	0.107542	1.961961	3.996	3.994	0.001471	1.076147	2.442	0
			EN	0.107509	0.961628	3.996	3.992	0.001453	1.060029	2.176	0
			COX	-	-	-	-	0.063020	49.758082	4	0
0.7	0.5	250	FULL	0.146416	1	0	0	0.044784	1	0	0
			ORACLE	0.136988	0.936427	4	0	0.003673	0.776682	4	0
			LASSO	0.128906	0.915496	3.978	3.984	0.005018	1.086479	2.488	0
			EN	0.128881	0.915515	3.984	3.986	0.004993	1.087609	2.328	0.004
			COX	-	-	-	-	0.027649	6.387825	4	0
		500	FULL	0.114623	1	0	0	0.002687	1	0	0
			ORACLE	0.110126	0.960421	4	0	0.00209	0.780737	4	0
			LASSO	0.105911	0.956897	3.922	3.988	0.002823	1.07776	2.52	0
			EN	0.105891	0.9570054	3.992	3.992	0.002813	1.073198	3.338	0
			COX	-	-	-	-	0.050620	21.109597	4	0
		1000	FULL	0.109052	1	0	0	0.001390	1	0	0
			ORACLE	0.106815	0.979529	4	0	0.001074	0.776022	4	0
			LASSO	0.140509	0.974029	3.992	3.996	0.001480	1.099749	2.38	0
			EN	0.104555	0.974431	3.992	3.994	0.001465	1.087278	2.12	0
			COX	-	-	-	-	0.065388	52.668729	4	0
0.7	0.6	250	FULL	0.216467	1	0	0	0.004091	1	0	0
			ORACLE	0.211372	0.976263	4	0	0.003230	0.789082	4	0
			LASSO	0.205515	0.959334	3.996	3.922	0.004265	1.067890	2.278	0
			EN	0.205482	0.959169	3.992	3.988	0.004238	1.064051	2.088	0
			COX	-	-	-	-	0.040094	10.976715	4	0
		500	FULL	0.152247	1	0	0	0.002168	1	0	0
			ORACLE	0.149122	0.979228	4	0	0.001712	0.789038	4	0
			LASSO	0.146747	0.974143	3.994	3.994	0.002296	1.092511	2.44	0
			EN	0.146781	0.974389	3.992	3.992	0.002190	1.047881	2.076	0
			COX	-	-	-	-	0.073076	37.771832	4	0
		1000	FULL	0.128311	1	0	0	0.001083	1	0	0
			ORACLE	0.126867	0.98866	4	0	0.000844	0.783138	4	0
			LASSO	0.125553	0.987048	3.988	3.988	0.001160	1.111553	2.218	0
			EN	0.125637	0.987610	3.994	3.988	0.001127	1.065074	1.97	0
			COX	-	-	-	-	0.093630	98.989981	4	0

## 6 An application to COVID-19 data

In this section, we demonstrate the utilisation of the penPHcure R package, which has been subject to local modification for the purpose of incorporating the EN penalty. This customised version has been named as “PenPHcure.AaRN". We demonstrate its functionality by applying it to the COVID-19 dataset comprising a sample of 19803 hospitalised patients in the Limpopo Province in South Africa. The COVID-19 data was obtained from the Department of Health. Given the involvement of human subjects, the research proposal was submitted to the Turfloop Research Ethics Committee (TREC) and ethical clearance was obtained. Since the study uses secondary data, informed consent was not applicable. The information on the participants has been treated with respect and dignity through protection of anonymity, while maintaining confidentiality. Gatekeeper permission for the use of the secondary data was sought from the Department of Health once the TREC approval letter was obtained. The data utilised in this study are available upon request through the National Health Research Database (NHRD) at https://nhrd.health.gov.za. The principal objective of this investigation was to explore the correlation between the duration from hospitalisation to death and various covariates observed throughout the follow-up period.

**Fig 4:**
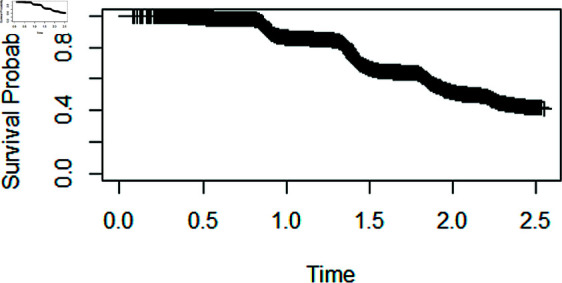
Kaplan-Meier plot.

Fig 4 shows the Kaplan-Meier plot, which exhibits a plateau at the end, signifying that there was sufficient follow-up. This plateau implies that the survival rates have reached a stable phase, suggesting the possibility of a substantial proportion being cured. The statistical Maller-Zhou test is performed to confirm the findings of the Kaplan-Meier plot.

The p-value associated with the Maller-Zhou test is approximately 1 . 845219*e* − 12. In this case, the p-value is extremely small, which implies that we reject the null hypothesis in favour of the alternative hypothesis and conclude that the follow up was sufficiently long.

### 6.1 COVID-19 data description

Table 5 outlines the crucial variables in our COVID-19 dataset. Each variable is carefully explained to ensure a clear comprehension of the various elements in the dataset.

**Table 5:** Covariates descriptions.

Variable	Description
PatientId	Identifier for each patient
DiagnosisProvince	Province where the diagnosis was made
Age	Age of the patient
Age.group	Age group to which the patient belongs
Sex	Gender of the patient
Birth.Date	Date of birth of the patient
Ethnic.Group	Ethnic group or race of the patient
Diagnosis.Date	Date of diagnosis
Admission.Date	Date of admission to the healthcare facility
LOS	Length of stay (LOS) in the healthcare facility
Facility	Name of the healthcare facility where the patient was admitted
FacilityType	Type of healthcare facility
Sector	Public or Private sector
District	District within the province
Sub.District	Sub-district within the district
OutcomeDate	Date of the patient’s outcome (e.g., recovery or death)
Healthcare.Worker	Indicates whether the patient is a healthcare worker (Yes or No)
Healthcare.Worker.Type	Type of healthcare worker
Ward.upon.admission	Ward type upon admission
Intensive.Care.Unit	Indicates whether the patient was admitted to the Intensive Care Unit (ICU)
High.Care	Indicates whether the patient was admitted to a high care unit
General.Ward	Indicates whether the patient was admitted to a general ward
Hypertension	Indicates whether the patient has hypertension (Yes, No, or Unknown)
Diabetes	Indicates whether the patient has diabetes (Yes, No, or Unknown)
Asthma	Indicates whether the patient has asthma (Yes, No, or Unknown)
Cardiac.Disease	Indicates whether the patient has a cardiac disease (Yes, No, or Unknown)
Chronic.Pulmonary.Disease	Indicates whether the patient has a chronic pulmonary disease (Yes, No, or Unknown)
Chronic.Renal.Failure	Indicates whether the patient has chronic renal failure (Yes, No, or Unknown)
Malignancy	Indicates whether the patient has malignancy (Yes, No, or Unknown)
HIV.Positive	Indicates whether the patient is HIV-positive (Yes, No, or Unknown)
Tuberculosis.Past	Indicates whether the patient had a past history of tuberculosis (Yes, No, or Unknown)
Tuberculosis	Indicates whether the patient has tuberculosis (Yes, No, or Unknown)
Obesity	Indicates whether the patient is obese (Yes, No, or Unknown)
Smoking	Smoking status of the patient
Pregnant	Indicates whether the patient is pregnant (Yes or No)
EverOxygenated	Indicates if the patient was ever administered oxygen
EverVentilated	Indicates if the patient was ever on a ventilator
AdmissionReason	Reason for admission

### 6.2 Models with no penalty

One of our objectives was to investigate what covariates play a role in explaining the probability of being cured (incidence) and the probability of being susceptible (latency) among COVID-19 patients. The glm for incidence and coxph for latency were used to check what variables are significant before the penalty was imposed. Coefficient values from both unpunished models are used as starting values in the penalised method.



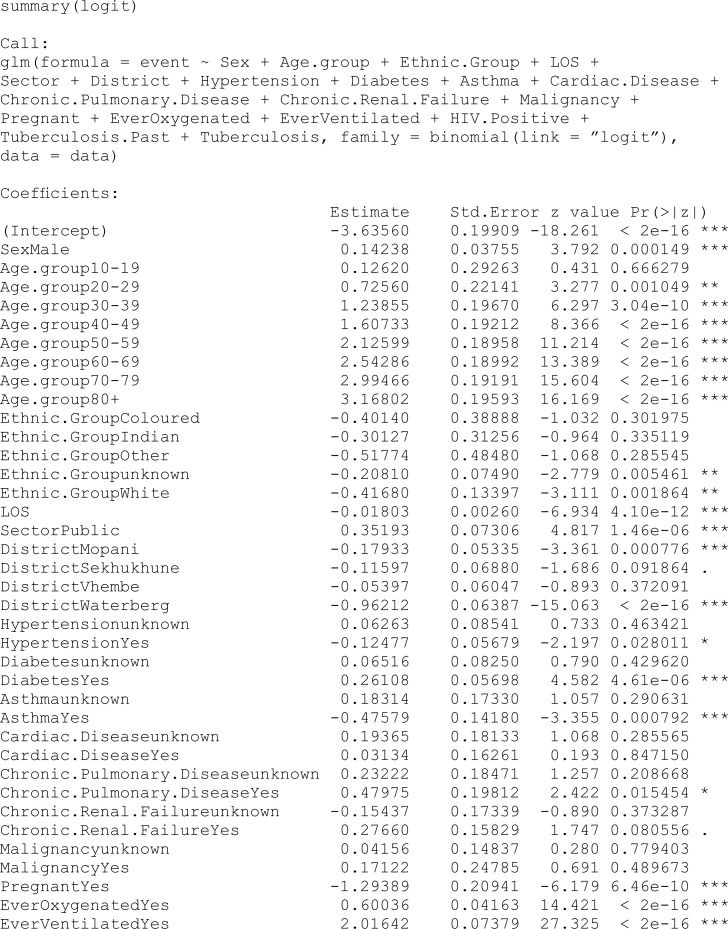





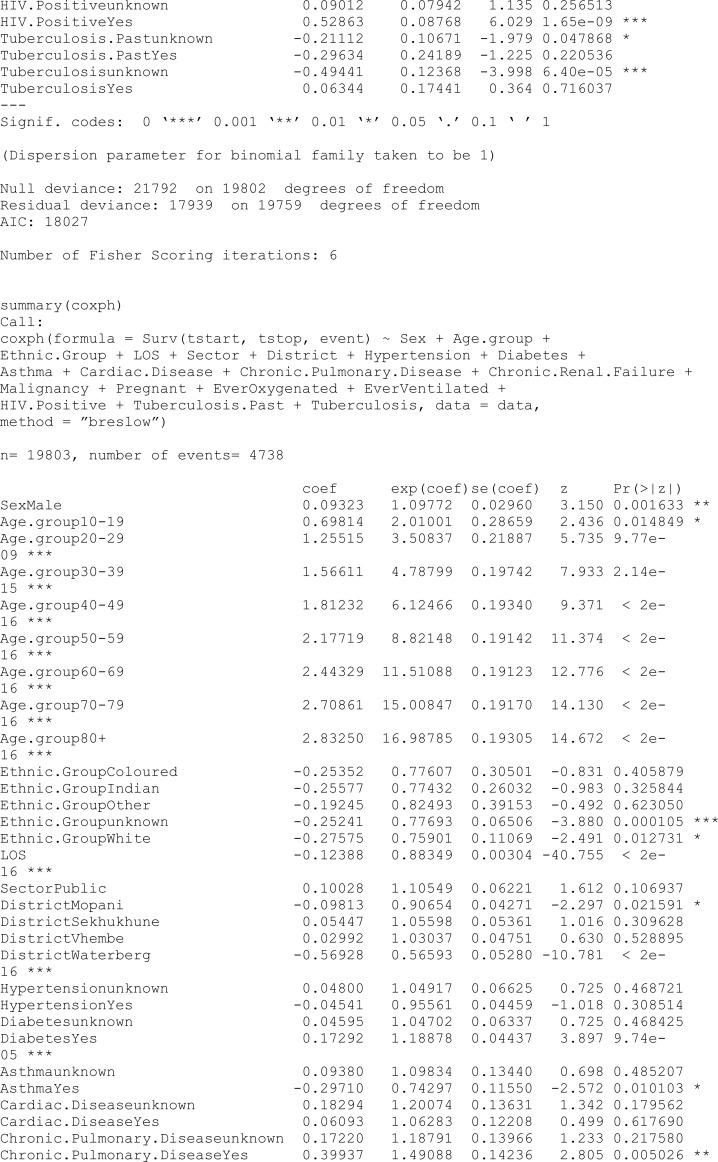





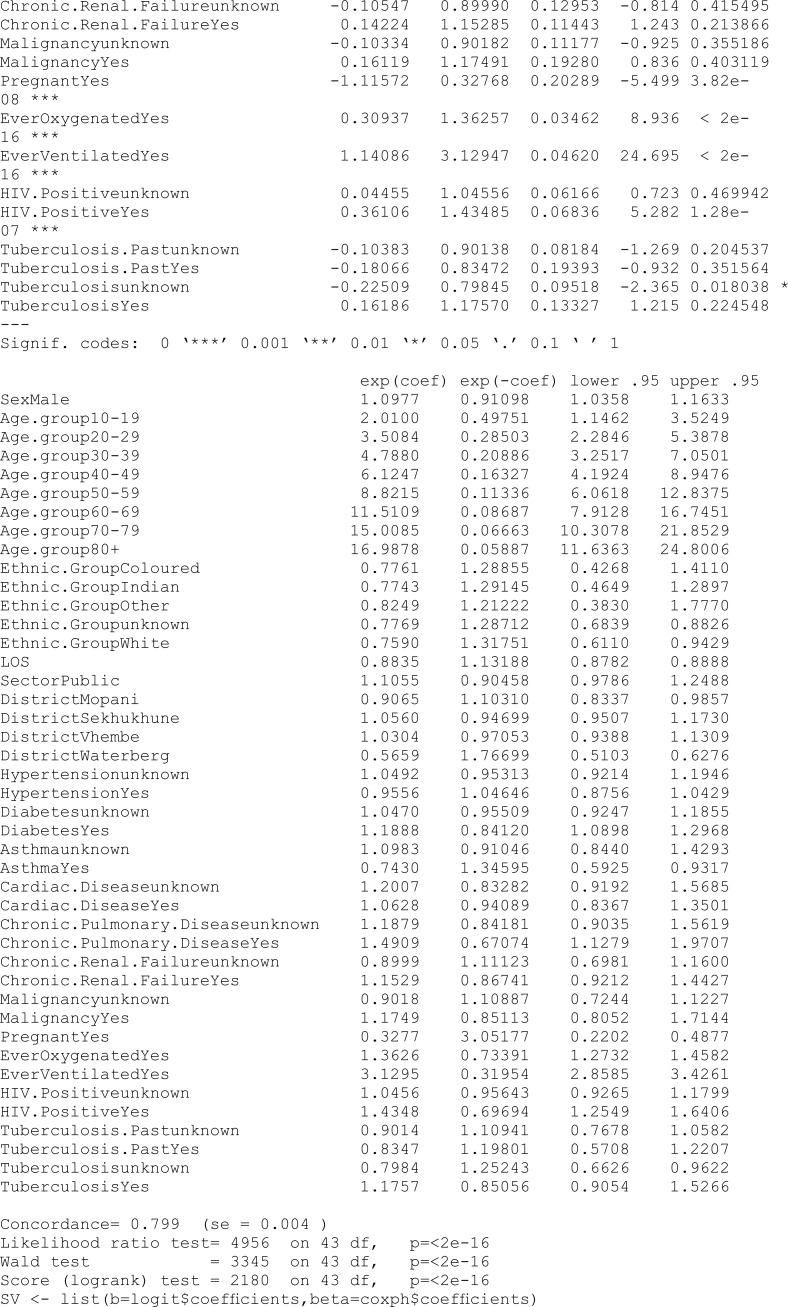



### 6.3 Mixture cure model with elastic net penalty

This section undertakes variable selection using the proposed EN-penalised likelihood method to explore the relevance of other covariates in explaining both incidence and latency. Initially, we designate the penalty type using the argument pen.type = "EN" and explore potential values for the tuning parameters (via the argument pen.tuneEN). Starting values are established based on the unpenalised models outlined earlier. A summary of the outcomes utilising the summary method is presented below, and the fitted model adhering to the lowest BIC is returned by default.



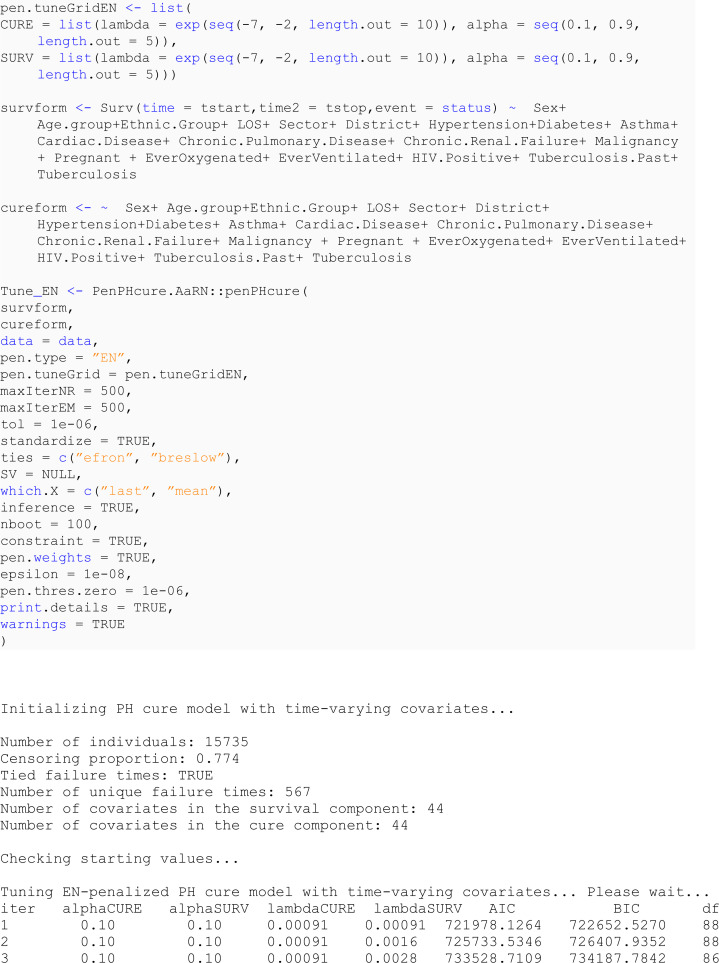





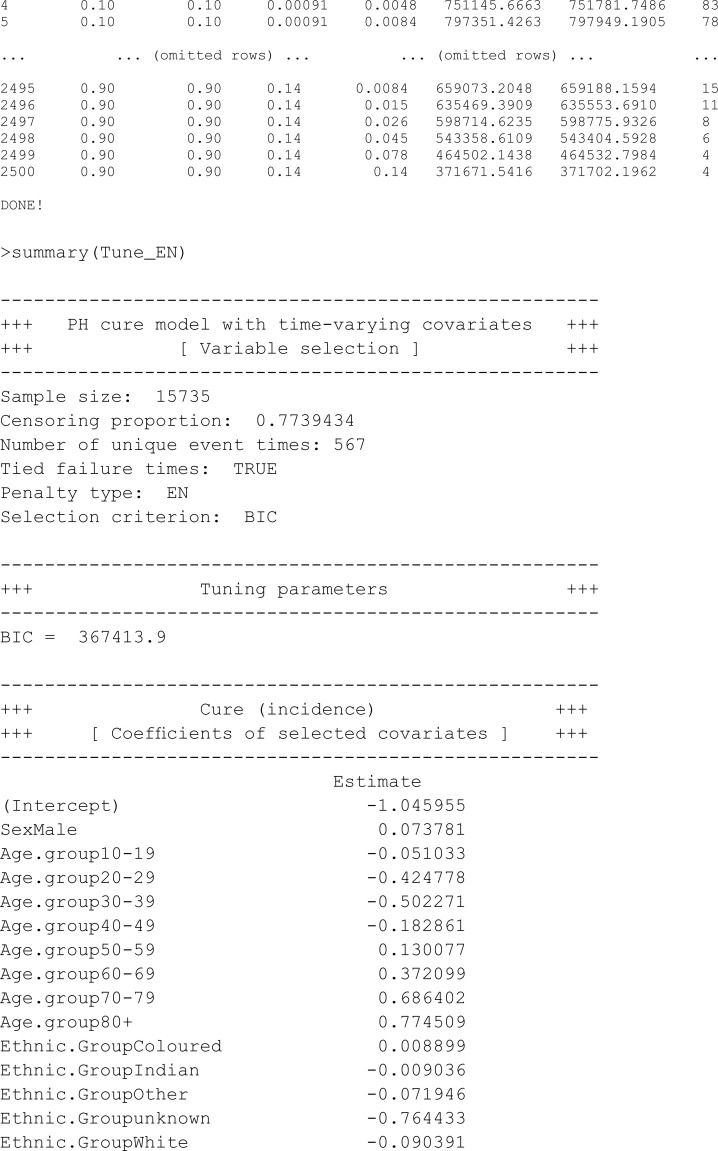





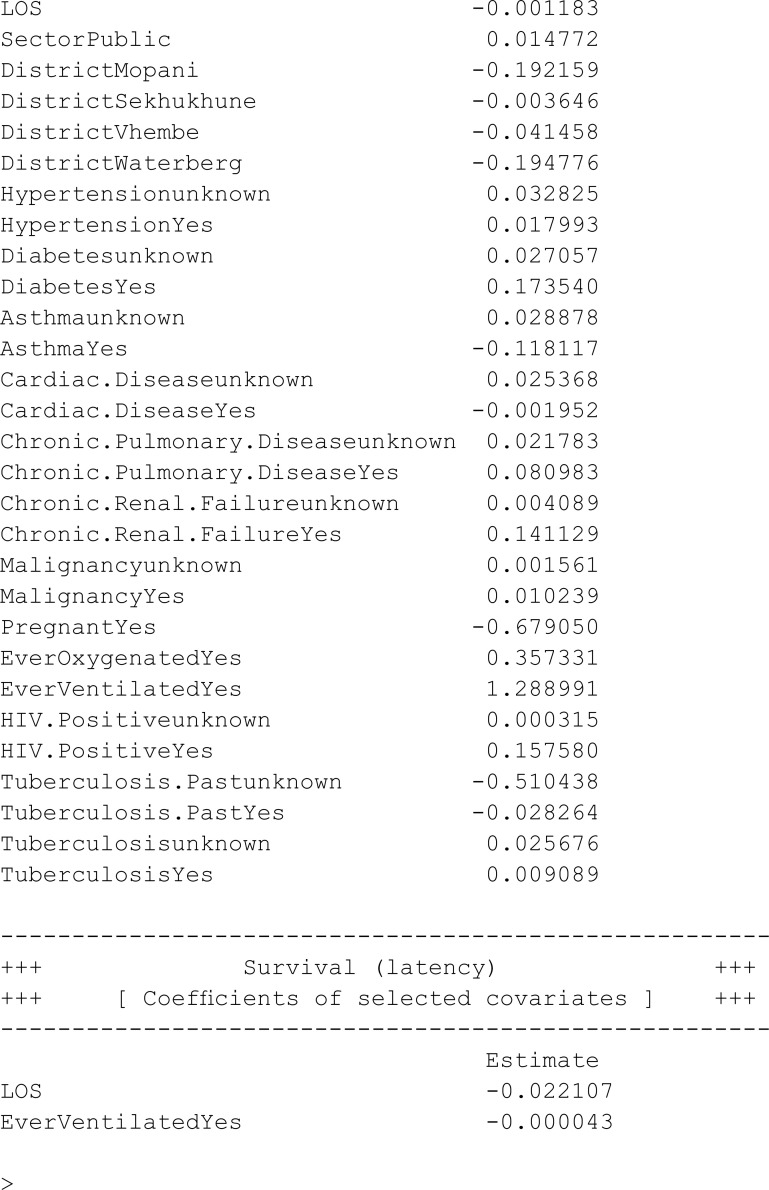



### 6.4 Interpretation of the results

In this study, the analysis focused on a PH cure model incorporating time-varying covariates. The dataset consisted of 15,735 samples, with a substantial censoring proportion of 77.39%. There were 567 unique event times, and tied failure times were observed, indicating instances of simultaneous occurrences. Variable selection was carried out using an EN penalty type, guided by the Bayesian information criterion (BIC). Tuning parameters for the cure (incidence) model were set at lambda = 0.008414677 and alpha = 0.9, while for the survival (latency) model, lambda was adjusted to 0.1353353, with an alpha of 0.5. The selection criterion, as measured by the BIC, resulted in a value of 470,708.8, reflecting the overall goodness of fit. These findings provide valuable insights into the model’s parameterisation, allowing for a nuanced understanding of the association between covariates and the observed outcomes in the context of COVID-19 hospitalisations in the specified region.

#### 6.4.1 Incidence (cure)

The incidence in the model represents the probability of being cured. A positive coefficient for a specific covariate implies that an increase in that covariate is associated with a higher likelihood of the event occurring, while a negative coefficient indicates that an increase in that covariate is linked to a lower likelihood of the event occurring.

The positive coefficient for males suggests that, on average, males have a higher likelihood of experiencing the event compared to females. In other words, being male is associated with an increased risk of the event.

On average, individuals aged 10 to 19 years, with a coefficient of –0.050133, individuals aged 20 to 29 years with a coefficient of –0.424778, individuals aged 30 to 39 years with a coefficient of –0.502271, and individuals aged 40 to 49 years with a coefficient of –0.182861 have a reduced likelihood of experiencing the event compared to the reference group (0 to 10 years old). Individuals aged 50 to 59 years, with a coefficient of 0.130077; individuals aged 60 to 69 years, with a coefficient of 0.372099; individuals aged 70 to 79 years, with a coefficient of 0.686402; and individuals aged 80 years and above, with a coefficient of 0.774509, have a greater likelihood of experiencing the event compared to those in the reference group (0 to 10 years old). Based on the coefficients, it seems that, as age increases, there is a corresponding increase in the likelihood of experiencing the event. The positive coefficients observed for age groups from “Age.group50-59” onward suggest a consistent trend of higher likelihood with older age. This pattern continues for subsequent age groups, with the growing coefficients indicating a progressively greater likelihood of the event.

The coefficients for various groups shed light on their associations with the likelihood of the event, with being Black as the reference group. Individuals from the Coloured ethnic group, as indicated by a coefficient of 0.008899, exhibit, on average, a higher likelihood of the event compared to their Black counterparts. In contrast, individuals belonging to the Indian ethnic group, with a coefficient of –0.009036, have a lower average likelihood of experiencing the event. Similarly, individuals categorised under “Other” ethnic groups, with a coefficient of –0.071946, have a lower average likelihood of the event compared to those in the Black reference group. The same trend is observed for individuals with an unknown ethnic group (coefficient: –0.764433) and those from the White ethnic group (coefficient: –0.090391), both displaying lower average likelihoods of the event in comparison to the reference group. In summary, these analyses suggest that, relative to individuals in the Black ethnic group, those in the Coloured ethnic group have a higher average likelihood of the event, while individuals in the Indian, Other, unknown, and White ethnic groups have lower average likelihoods.

On average, for each unit increase in the length of stay (LOS), the likelihood of the event decreases by a factor of –0.001183. In simpler terms, a longer LOS is associated with a slightly lower average likelihood of experiencing the event. The negative coefficient suggests an inverse relationship, implying that, as the LOS increases, the risk or likelihood of the event decreases.

On average, individuals in the public sector, as represented by the coefficient of 0.014772, have a higher likelihood of experiencing the event, compared to their counterparts in the private sector (the reference group). The positive coefficient indicates that, relative to the private sector, individuals in the public sector exhibit an increased average likelihood of experiencing the event.

On average, individuals in the Mopani, Sekhukhune, Vhembe, and Waterberg Districts, as indicated by the coefficient of –0.192159, –0.003546, –0.041458, and –0.194776 respectively, have a reduced likelihood of the event compared to those in the Capricorn District.

Individuals with unknown hypertension status, as denoted by a coefficient of 0.032825, demonstrate, on average, a higher likelihood of the event compared to those without hypertension. Similarly, individuals with diagnosed hypertension, represented by a coefficient of 0.017993, show, on average, a higher likelihood of the event compared to those without hypertension. The positive coefficient suggests that individuals with hypertension, on average, display an increased average likelihood of experiencing the event compared to their counterparts without hypertension.

Individuals with unknown diabetes status, as indicated by a coefficient of 0.027057, demonstrate, on average, a higher likelihood of the event compared to those without diabetes. Similarly, individuals with diagnosed diabetes, represented by a coefficient of 0.173540, display, on average, a higher likelihood of the event when compared to those without diabetes. The positive coefficient implies that individuals with diabetes, on average, show an increased average likelihood of experiencing the event compared to their counterparts without diabetes. In summary, when compared to individuals without diabetes, both those with unknown diabetes status and those diagnosed with diabetes have higher average likelihoods of the event.

Individuals with unknown asthma status, as denoted by a coefficient of 0.028878, demonstrate, on average, a higher likelihood of the event compared to those without asthma. The positive coefficient suggests that, in relation to individuals without asthma, those with unknown asthma status exhibit an increased average likelihood of experiencing the event. Conversely, individuals with diagnosed asthma, represented by a coefficient of –0.118117, display, on average, a lower likelihood of the event compared to those without asthma. The negative coefficient implies that individuals with asthma, on average, show a decreased average likelihood of experiencing the event compared to their counterparts without asthma.

Individuals with unknown cardiac disease status, denoted by a coefficient of 0.025368, exhibit, on average, a higher likelihood of experiencing the event compared to those without cardiac disease. The positive coefficient suggests that, relative to individuals without known cardiac disease, those with unknown cardiac disease status show an increased average likelihood of experiencing the event. Conversely, individuals with diagnosed cardiac disease, represented by a coefficient of –0.001952, display, on average, a lower likelihood of the event compared to those without cardiac disease. The negative coefficient implies that individuals with cardiac disease, on average, show a decreased average likelihood of experiencing the event compared to their counterparts without cardiac disease.

Individuals with unknown chronic pulmonary disease status, as denoted by a coefficient of 0.021783, exhibit, on average, a higher likelihood of the event compared to those without chronic pulmonary disease. The positive coefficient suggests that, relative to individuals without chronic pulmonary disease, those with unknown status show an increased average likelihood of experiencing the event. Conversely, individuals with diagnosed chronic pulmonary disease, represented by a coefficient of 0.080983, display, on average, a higher likelihood of the event compared to those without chronic pulmonary disease. The positive coefficient indicates that individuals with chronic pulmonary disease, on average, show an increased average likelihood of experiencing the event compared to their counterparts without chronic pulmonary disease.

Individuals with unknown chronic renal failure status, as denoted by a coefficient of 0.004089, display, on average, a higher likelihood of the event compared to those without chronic renal failure. The positive coefficient suggests that, relative to individuals without known chronic renal failure, those with unknown status show a increased average likelihood of experiencing the event. Conversely, individuals with diagnosed chronic renal failure, represented by a coefficient of 0.141129, exhibit, on average, a higher likelihood of the event compared to those without chronic renal failure. The positive coefficient indicates that individuals with chronic renal failure, on average, show an increased average likelihood of experiencing the event compared to their counterparts without chronic renal failure.

Individuals with unknown malignancy status, as indicated by a coefficient of 0.001561, display, on average, a slightly higher likelihood of the event compared to those without malignancy. The positive coefficient suggests that, relative to individuals without malignancy, those with unknown status show a modestly increased average likelihood of experiencing the event. Conversely, individuals with diagnosed malignancy, represented by a coefficient of 0.010239, exhibit, on average, a higher likelihood of the event compared to those without malignancy. The positive coefficient indicates that individuals with malignancy, on average, show an increased average likelihood of experiencing the event compared to their counterparts without malignancy. In summary, when compared to individuals without malignancy status, those with unknown status have a slightly higher average likelihood of the event, while those with diagnosed malignancy have a higher average likelihood.

Individuals identified as pregnant, as indicated by a coefficient of –0.67050, demonstrate, on average, a significantly lower likelihood of the event compared to their non-pregnant counterparts. The negative coefficient implies that, relative to non-pregnant individuals (the reference group), pregnant individuals exhibit a substantial decrease in the average likelihood of experiencing the event.

Individuals who were oxygenated at any point in their treatment, as indicated by a coefficient of 0.3573331, demonstrate, on average, a higher likelihood of experiencing the event compared to those who were never oxygenated. The positive coefficient suggests that, relative to individuals without a history of oxygen therapy, those with such a history exhibit an increase in the average likelihood of experiencing the event. It should be noted that patients who received oxygen were severely ill, and the probability of their mortality might not be directly related to the usage of oxygen.

Individuals who were ventilated at any point in their treatment, as indicated by a coefficient of 1.288991, demonstrate, on average, a substantially higher likelihood of the event compared to those who were never ventilated. The large positive coefficient suggests that, relative to individuals without a history of ventilation, those who have been ventilated show a significant increase in the average likelihood of experiencing the event. It should be noted that patients who were ventilated were severely ill, and the probability of their mortality might not be directly related to the usage of ventilation machine.

Individuals with unknown HIV status, as indicated by a coefficient of 0.000315, exhibit, on average, a slightly higher likelihood of the event compared to those who are not HIV positive. The positive coefficient suggests that, relative to individuals without HIV status, those with unknown status show a modest increase in the average likelihood of experiencing the event. Conversely, individuals with diagnosed HIV, represented by a coefficient of 0.15780, demonstrate, on average, a higher likelihood of experiencing the event compared to those who are not HIV positive. The positive coefficient indicates that individuals with HIV, on average, exhibit a notable increase in the average likelihood of experiencing the event compared to their counterparts without HIV.

Individuals with unknown past tuberculosis, as indicated by a coefficient of –0.510438, display, on average, a lower likelihood of experiencing the event as compared to those without a past history of tuberculosis. The negative coefficient suggests that, relative to individuals without past tuberculosis status, those with unknown status show a decreased average likelihood of experiencing the event. Conversely, individuals with a past history of tuberculosis, represented by a coefficient of –0.0282264, demonstrate, on average, a lower likelihood of the event as compared to those without a past history. The negative coefficient indicates that individuals with past tuberculosis, on average, exhibit a decrease in the average likelihood of experiencing the event when compared to their counterparts without a past history.

Individuals with unknown current tuberculosis status, as denoted by a coefficient of 0.025676, exhibit, on average, a higher likelihood of the event as compared to those without current tuberculosis. The positive coefficient suggests that, relative to individuals without known current tuberculosis status, those with unknown status show an increased average likelihood of experiencing the event. Similarly, individuals currently diagnosed with tuberculosis, represented by a coefficient of 0.009089, demonstrate, on average, a higher likelihood of the event when compared to those without current tuberculosis. The positive coefficient indicates that individuals with current tuberculosis, on average, exhibit an increase in the average likelihood of experiencing the event compared to their counterparts without current tuberculosis.

#### 6.4.2 Latency (survival)

The coefficients in the latency component offer insights into the impact of various covariates on the survival or latency time of individuals not cured. Specifically, a positive coefficient for a covariate indicates an elevated hazard rate for individuals yet to experience the event, signifying that as the covariate value rises, the risk of the event increases. On the other hand, a negative coefficient signals a diminished hazard rate, implying that an increase in the covariate value is linked to a reduced risk of the event. These coefficients shed light on how specific factors influence the instantaneous risk for individuals who have not yet encountered the event, with a positive coefficient suggesting an increased risk and a negative coefficient indicating a decreased risk associated with the respective covariate.

The coefficient for LOS (length of stay) was estimated at –0.022107. This coefficient indicates the impact of LOS on the hazard of the event, with a negative value suggesting that, on average, a longer LOS is associated with a lower hazard of the event. In other words, patients with a longer duration of hospitalisation tend to have a slightly decreased risk of event of interest.

Patients who have undergone ventilation, as indicated by a coefficient estimate of –0.00043, generally experience a significantly lower hazard of the event when compared to those who have not been ventilated.

## 7 Discussion

In order to rigorously assess the efficacy of the developed penalised logistic/Cox PH mixture cure model, a comprehensive simulation study was conducted. The model, adeptly designed to handle both the cure status with time-varying covariates and the survival of uncured subjects with time-varying covariates using an EN penalty, underwent meticulous evaluation. In our simulations, we explore six (6) simulation scenarios, each characterised by varying levels of censoring and proportions of individuals cured. These proportions are presented as fractions of the sample size and are determined by specific values assigned to each scenario. Our investigation encompasses three distinct sample sizes, namely: *N* = 250 , 500 , 1000. For each setting, we conducted 500 replications using a dataset we constructed, incorporating time-varying covariates for both latency and incidence.

The results of these simulations revealed compelling evidence of superior performance, with the proposed model consistently demonstrating smaller errors when compared to the previously proposed model utilising LASSO. The mean estimation errors for logistic/Cox proportion hazards via an EN penalty with time-varying covariates for both latency and incidence were consistently lower compared to when the LASSO penalty was used. This compelling evidence leads us to the conclusion that the adoption of an EN within the framework of the mixture cure model demonstrates superior performance compared to its LASSO counterpart.

This outcome underscores the robustness and effectiveness of the EN regularisation in enhancing the precision and predictive accuracy of our developed model. The observed performance gains do not only validate the significance of the introduced modifications, but also position our model as a promising advancement in the field of survival analysis.

## 8 Conclusion

Before using the COVID-19 dataset, the existence of a cure fraction was determined. The Kaplan-Meier plot exhibits a plateau at the end, signifying that there was sufficient follow-up. This plateau implies that the survival rates have reached a stable phase, suggesting the possibility of a substantial proportion being cured.

In the context of our analyses, we present a summary of the key findings and contributions of the proposed model. The proposed model enhanced prediction accuracy by effectively shrinking or eliminating less significant coefficients. While this process introduces a minor degree of bias, it concurrently reduced the variance in the projected values, leading to an overall improvement in prediction accuracy. By shrinking some coefficients and setting others to zero, the proposed model retained only the important variables, achieving parsimony. Adding a penalty to the cure model reduces error and avoids overfitting.

The proposed model successfully discerns significant covariates linked to the survival of COVID-19 patients, arising from the process of shrinking or excluding unimportant variables in the model. To demonstrate the application of the proposed model, a real dataset was used, examining the time until death for patients hospitalised due to COVID-19 in the Limpopo Province. Below are the key findings from utilising the logistic/Cox PH mixture cure model with an elastic net penalty. These findings provide valuable insights into the factors influencing both the incidence of cure and latency, thus contributing to a comprehensive understanding of survival outcomes for individuals under consideration.

### 8.1 Incidence (cure) component

All covariates were not removed for incidence. Males have a higher likelihood of the event compared to females. Older age groups show an increasing likelihood of the event, with a consistent trend of higher risk for individuals aged 50 years and above. Coloured individuals have a higher average likelihood, while Indian, Other, unknown, and White ethnic groups have lower average likelihoods compared to the Black reference group.

Longer LOS is associated with a slightly lower average likelihood of the event. Individuals in the public sector exhibit a higher average likelihood compared to those in the private sector. Individuals in specific districts (Viz., Mopani, Sekhukhune, Vhembe, and Waterberg) have a reduced likelihood compared to those in Capricorn District.

For hypertension, diabetes, asthma, cardiac disease, chronic pulmonary disease, chronic renal failure, and malignancy, known cases exhibit higher average likelihoods than unknown cases or those without the respective conditions. Pregnant individuals show a significantly lower average likelihood compared to non-pregnant individuals.

Oxygenated and ventilated individuals have higher average likelihoods, but the association may be confounded by the severity of illness. Known HIV cases exhibit a higher average likelihood compared to unknown cases or those without HIV. Past tuberculosis is associated with a lower average likelihood, and current tuberculosis is linked to a higher average likelihood.

### 8.2 Latency (survival) component

For latency, most covariates were removed from the model except LOS at hospital and ventilation status. Longer LOS was associated with a lower hazard of the event. Ventilated patients generally experience a significantly lower hazard of the event when compared to non-ventilated individuals.

## 9 Future research directions

The findings of this study provide possible areas for further research in the future. The following possible future research directions are suggested:

- One possible direction for future research may include the use of single index model/Cox PH model using a penalty. Single index models offer an efficient approach to reduce dimensionality and circumvent the challenges posed by the “curse of dimensionality” encountered in multivariate non-parametric regression.
- Understanding the separate impacts of significant covariates can be a difficult task. In various scenarios, researchers might desire to assess the influence of certain critical covariates on the likelihood of an outcome, while simultaneously maintaining a flexible modelling approach for the variable p (x). In extensive clinical studies, certain covariates may be considered as “nuisance" variables, while others hold great importance to the researcher. It would be beneficial to have the best of both worlds, combining the interpretability of a logistic model with the flexibility of a single-index model. One may want to use the Generalised Partial-Linear Single-Index Model imposing a penalty.

In essence, the ongoing exploration for improved and reliable statistical techniques in survival analysis, incorporating penalties, remains crucial for the continual refinement and selection of significant covariates.
